# Measuring health literacy and its associations with health behaviors of adolescents in Germany

**DOI:** 10.1093/eurpub/ckac129.319

**Published:** 2022-10-25

**Authors:** A Loer, OM Domanska, S Jordan

**Affiliations:** Department of Epidemiology and Health Monitoring, Robert Koch Institute, Berlin, Germany

## Abstract

**Background:**

The questionnaire “Measurement of Health Literacy Among Adolescents Questionnaire” (MOHLAA-Q) was developed and validated in a multi-stage process to measure generic health literacy among 14- to 17-year-olds. The MOHLAA-Q combines subjective and objective measurements, consisting of four scales. The instrument was applied to explore associations between generic health literacy and different health behaviors among adolescents in Germany. The data should contribute to the age-appropriate development of health promotion interventions.

**Methods:**

We carried out a nationwide cross-sectional online survey with 1,235 adolescents aged 14-17 years in Germany in 2019. Data on generic health literacy were collected using the four scales of the MOHLAA-Q and data on health behaviors were collected using single established single-item questions on sports, diet, alcohol consumption, and smoking. Bivariate and multiple analyses were performed for investigating associations between generic health literacy and health behaviors, adjusted for age, gender, education and self-efficacy.

**Results:**

Not consuming fruit and vegetables daily was associated with lower health literacy levels in all examined scales. Doing no sports as well as smoking showed positive associations with low levels in “health-related communication and interaction skills” and “attitudes toward one's own health and health information”. No associations were found between risky alcohol consumption and health literacy.

**Conclusions:**

The results indicate that strengthening health literacy should be part of health promotion activities for increasing physical activity and healthy diet among adolescents. The MOHLAA-Q allows us to identify which specific dimensions of health literacy might be addressed in order to promote different health behaviors.

